# A Case of COVID-19-Associated Pediatric Multisystem Inflammatory Syndrome in Shock Managed by Cytokine Filtration

**DOI:** 10.1155/2022/3373289

**Published:** 2022-02-02

**Authors:** Priyanka Lalwani, Subashini Baskaran, Diego Arango Uribe, Anil Ramaiah, Aqdas Saqib, Mohammed ElMesserey, Emad M. Fathi, Yuichi Tabata, Christoph Fink, Marco Pallavidino

**Affiliations:** ^1^Pediatric Intensive Care Unit, Al Jalila Children's Specialty Hospital, Dubai, UAE; ^2^Mohammed Bin Rashid University Of Medicine and Health Sciences, Dubai, UAE

## Abstract

Multisystem inflammatory syndrome in children (MIS-C) after COVID-19 has been recognized as a complication arising due to cytokine storm. Several management strategies including intravenous immunoglobulin and immunomodulators have been reported. This case report highlights the use of a cytokine filter (oXiris®) in the management of MIS-C. Cytokine filters eliminate cytokines and reduce the demand for vasopressors in patients with other inflammatory conditions. A 7-year-old child with prolonged fever, vomiting, hypotension, elevated inflammatory mediators, and dilatation of coronary arteries on echocardiography was found to have positive SARS-CoV-2 IgG and PCR. He was diagnosed as MIS-C and was managed in the pediatric intensive care unit. He required ventilatory support, vasopressors, and continuous renal replacement therapy (CRRT) with a cytokine filter. He showed marked improvement within 24 hours of initiating CRRT. Cytokine filters may have a potential role in the management of severely ill children due to MIS-C. To our knowledge, this is the first report of successful use of the oXiris^®^ membrane in MIS-C. However, further case series and controlled trials are needed to establish its use in this condition.

## 1. Introduction

Multisystem inflammatory syndrome in children (MIS-C) is a postinfectious complication of COVID-19. MIS-C is suspected in children with fever and involvement of two or more organ systems, who have an evidence of SARS-CoV-2 infection by PCR, serology, or antigen test or have an exposure to a suspected or confirmed COVID-19 case within the past 4 weeks. Gastrointestinal and cardiovascular involvement is seen in the majority of patients along with elevation of inflammatory markers such as C-reactive protein (CRP), procalcitonin, ferritin, erythrocyte sedimentation rate (ESR), and D-dimer [1].

The current literature indicates that the pathophysiology of MIS-C is mainly due to dysregulated inflammatory response, also known as cytokine storm, in addition to direct viral damage. Viral infection triggers uncontrolled production of proinflammatory mediators such as interleukin-1 (IL-1), interleukin-6 (IL-6), and tumor necrosis factor (TNF-*α*), which contribute to the disease severity [1, 2]. This pattern is also seen in other similar conditions such as Kawasaki shock syndrome, sepsis, ARDS, and macrophage activation syndrome (MAS).

Recent evidence supports the use of high-dose intravenous immunoglobulin (IVIG) at 2 grams/kilogram and methylprednisolone in the management of MIS-C. The use of biologic agents such as anakinra and tocilizumab is being practiced in refractory cases [3].

Cytokine filters have been used in patients with life-threatening emergencies that are associated with dysregulated immune response such as septic shock, ARDS, and severe acute respiratory infection due to influenza. Studies have demonstrated that hemodynamic stabilization and reversal of shock, including improvement in lactate clearance, can be achieved by its use [4]. To our knowledge, the use cytokine filters has not been reported in MIS-C.

## 2. Case Report

We discuss a case of a 7-year-old, previously healthy boy who was transferred to our facility for features of septic shock. He was admitted to the referring hospital with a history of fever, vomiting, and diarrhea of 5 days. He had raised inflammatory markers and sterile cultures, and COVID-19 PCR was negative. He was empirically started on broad-spectrum antibiotics. Since his condition deteriorated rapidly with profound respiratory distress, he was intubated and started on mechanical ventilation. Meanwhile, fluid resuscitation and vasopressors were given for hypotension. Cardiac enzymes were deranged, and echocardiography revealed dilated coronary arteries. Serum amylase and lipase were elevated, and abdominal ultrasound showed mild hepatomegaly, mesenteric edema, and adenopathy in the right lower quadrant. Hence, MIS-C was suspected, and he was started on IVIG (2 g/kg) and pulse steroid therapy with high-dose methylprednisolone 30 mg/kg for 3 days.

After this initial management, he was transferred to our pediatric intensive care unit. Repeat testing for SARS-CoV-2 by PCR and serology (IgG) was positive. He continued to be in severe distributive shock requiring high doses of epinephrine (0.3 mcg/kg/min) and norepinephrine (0.3 mcg/kg/min) to maintain a mean arterial pressure of 60–65 mmHg. Inflammatory markers, interleukins, and cardiac enzymes were all grossly elevated. A white blood cell count of 13,000/*μ*l (reference range: 5000–13000/*μ*l), CRP 288 mg/L (reference range: <2.8 mg/L), procalcitonin 35 ng/ml (reference range: <0.5 ng/ml), lactate 1.7 mmol/L (reference range: <1.6 mmol/L), D-dimer 5.02 ug/ml (reference range: <0.5 ug/ml), fibrinogen 474 mg/dl (reference range: <409 mg/dl), ferritin 1132 ng/ml (reference range: 14–124 ng/ml), IL-6 3725 pg/ml (reference range: <7), albumin 1.7 g/dl (reference range: 3.8–5.4 g/dl), pro-BNP 7426 pg/ml, and troponin-I 2.8 ng/ml (reference range: <0.04 ng/ml) were seen. Chest X-ray reported left lower lobe consolidation, and echocardiography revealed dilated coronary arteries with the left main coronary artery being most severely affected, which was beginning to become aneurysmal (5 mm; Z-score +4.3). In addition, there was severe hypokinesia of the intraventricular septum along with mild pericardial effusion. In view of low cardiac output due to myocardial dysfunction, continuous renal replacement therapy (CRRT) using cytokine filter was initiated. Meanwhile, steroid therapy was continued, and the child received systemic anticoagulation.

**Figure 1:**
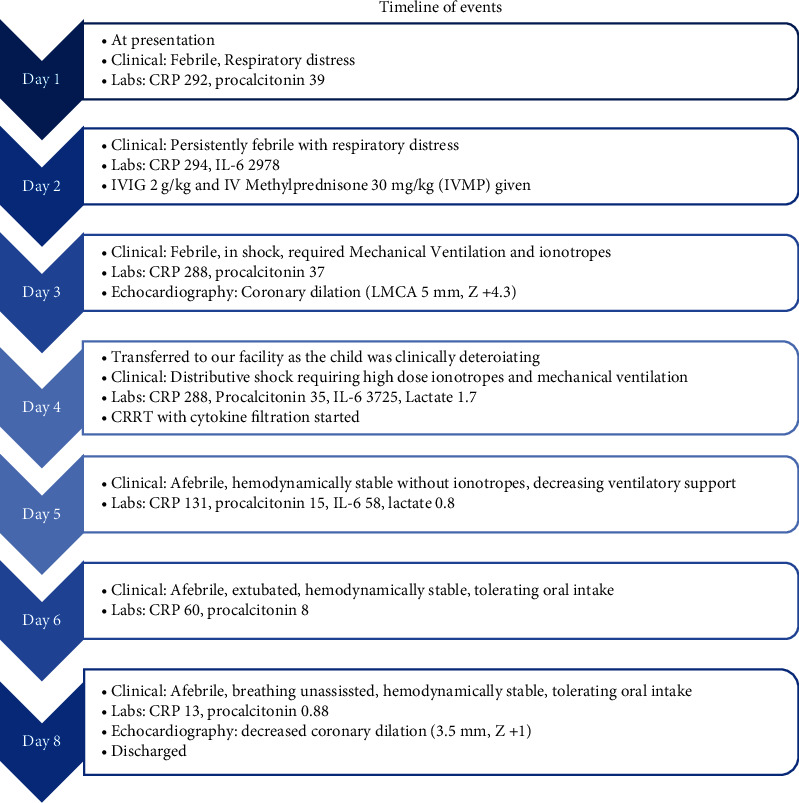
Timeline of events.

**Figure 2:**
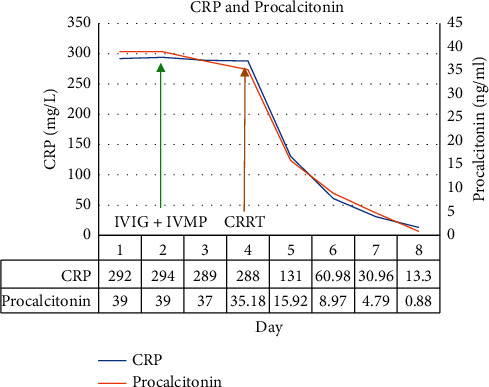
Trend of CRP and procalcitonin.

**Figure 3:**
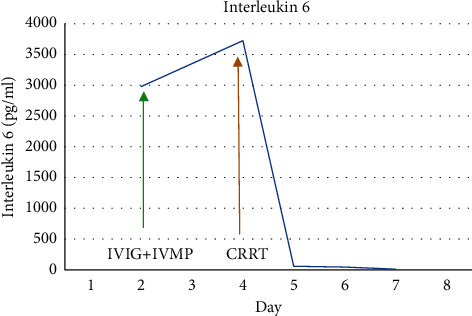
Trend of interleukin 6 (IL-6).

**Figure 4:**
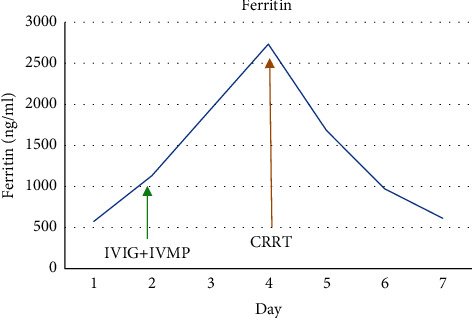
Trend of ferritin.

**Figure 5:**
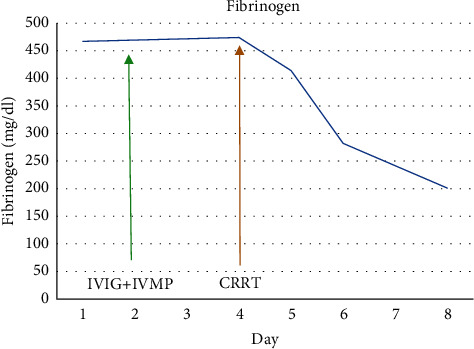
Trend of fibrinogen.

**Figure 6:**
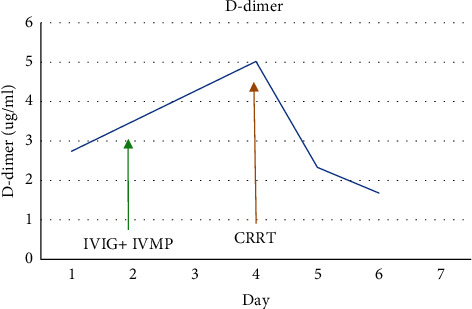
Trend of D-dimer.

There was a significant improvement in the clinical condition of the child over the next 24 hours. Within 8 hours after initiation of CRRT, IL-6 decreased from 3725 pg/ml to 58.6 pg/ml and within 24 hours, and CRP and procalcitonin halved. The child became afebrile, hemodynamically stable without inotropic support, and tolerated trophic feeds. CRRT with cytokine filter was continued for a total of 48 hours along with high-dose methylprednisolone. Serial CRP, procalcitonin, lactate, ferritin, fibrinogen, IL-6, and troponin-I were monitored daily during the hospital stay. All inflammatory markers showed a steady decline (Figures 1–6). After 48 hours of CRRT, he was successfully extubated and was breathing on room air within 24 hours after extubation. Chest X-ray showed improvement in lung infiltration.

He required intensive care for three days and was discharged from the hospital within a week. At the time of discharge, his COVID-19 PCR was reported negative, and repeat echocardiogram showed remarkable changes such as decreased coronary dilation (3.5 mm, *Z* + 1), improved cardiac function, and resolved pericardial effusion. He was prescribed low-dose aspirin and esomeprazole at the time of discharge.

## 3. Discussion

MIS-C associated with SARS-CoV-2 is being increasingly recognized worldwide. This syndrome is characterized by excessive production of proinflammatory cytokines including tumor necrosis factor (TNF), interleukin-6 (IL-6), and interleukin-1 (IL-1) resulting in symptoms of high fever, rashes, coagulopathy, prominent gastrointestinal, and cardiac changes with some progressing to multiple organ failure and death [3]. A high index of suspicion is required to make the diagnosis. Once diagnosed, ill-appearing patients should be stabilized and transferred to a tertiary-care unit as they can deteriorate rapidly [5, 6].

Initial management usually includes IVIG 2 g/kg, corticosteroids, and anticoagulation as seen in our case. Immunomodulators such as anakinra (recombinant human IL‐1 receptor antagonist) and tocilizumab (IL-6 receptor antagonist) have also been used in refractory cases as this syndrome displays characteristics similar to macrophage activation syndrome (MAS) [3]. The usual time for clinical response after IVIG and methylprednisone is up to 48 hours. The resolution of fever and decreasing inflammatory markers allow for tapering of steroids. However, in a minority of patients, there is no clinical response; hence, these cases require a second-line agent such as anakinra or tocilizumab [3, 7]. In this case, cytokine filtration was performed instead of giving immunomodulators as the child was clinically worsening despite the first-line management.

The presumed pathophysiology of MIS-C is dysregulated inflammatory response leading to cytokine storm. The rationale of using CRRT with a cytokine filter is to eliminate the proinflammatory cytokines as seen in MIS-C. Extracorporeal organ support therapies including hemoperfusion, with efficient sorbent cartridges for removal of cytokines and other circulating inflammatory mediators, have been well reported in critically ill patients [8]. The ideal time to start CRRT with a cytokine filter is debatable. However, in a retrospective study, it was observed that the preferred timing of initiating the cytokine filtration would be before the signs of multiorgan dysfunction are observed [9].

The cytokine filter used in this case was an oXiris membrane which is an AN69 membrane with the surface treated with polyethyleneimine and grafted with heparin. The AN69 membrane enables adsorption of cytokines, and the polyethyleneimine surface treatment allows for the adsorption of endotoxins while providing renal support by diffusion and convection. The membrane effectively removes endotoxin, TNF-*α*, IL-6, IL-8, and IFN*γ* [10].

This membrane has been beneficial in severe septic shock patients requiring ionotropic support and has proven to decrease ionotropic demand and reduce cytokines [10, 11]. The cytokine filtration is found to be more beneficial if started earlier in the course of illness [9]. There was a significant reduction in inflammatory mediators including IL-6 and lactate in a randomized double-blinded trial conducted on patients with sepsis [10]. We observed similar reduction in inflammatory mediators and lactate within 24 hours of filtration.

The requirement for vasopressors in MIS-C has been reported to be for up to 10 days. Coronary artery dilatation has been reported to regress by 30 days [2]. With our case, vasopressors could be weaned off completely within 24 hours of starting CRRT with a cytokine filter. In addition, we observed early regression of the coronary artery dilatation as the *Z* score reduced by three within a week. We believe the substantial improvement in the clinical status and inflammatory mediators along with myocardial recovery in a short time frame was likely due to the use of cytokine filter in our patient.

Our experience could provide a valuable insight to a potential management strategy which could be used in severely ill patients diagnosed with MIS-C. In addition to hastening the recovery, it could prevent some of the devastating sequelae of this condition, such as the coronary artery aneurysms. Cytokine filters have been used extensively in other conditions such as septic shock and ARDS, but to our knowledge, this is the first report of such usage in MIS-C. However, we acknowledge that this is a single-case experience, and a case series with good number of patients and eventually controlled trials would be needed before making recommendation regarding the same.

## Data Availability

The data used to support the findings of this study are available from the corresponding author upon request.
